# Antiphospholipid Antibody Induced by Nivolumab

**DOI:** 10.1155/2018/3106852

**Published:** 2018-01-11

**Authors:** Ahmed Aburahma, Nour Aljariri Alhesan, Farah Elounais, Emad Abu Sitta

**Affiliations:** Hurley Medical Center, Michigan State University, East Lansing, MI, USA

## Abstract

Nivolumab is a monoclonal antibody against the programmed death protein 1 and is used for patients with advanced melanoma. It is associated with potentially immune-related adverse events, including disorders of the skin, GI tract, and the thyroid; these disorders were successfully treated with prednisone and infliximab. Other immunotherapeutic agents were observed to induce the formation of antiphospholipid antibody (APA) including *α*-interferon and interleukin-2. We present a case of APA development after the third dose of nivolumab in a 71-year-old male with advanced melanoma. The APA was detected after finding a prolonged aPTT; the lupus anticoagulant assay tested positive. The patient was treated with prednisone but, unfortunately, he expired a few days later.

## 1. Background

Nivolumab is a monoclonal antibody against the programmed death protein 1 (PD-1) that is used mainly in treating advanced melanoma and non-small cell lung cancer [1, 2]. The most common adverse effects reported for nivolumab are fatigue, rash, and diarrhoea; however, a few other rare side effects involving mainly the GI tract, lung, and skin have been reported and are thought to be related to the immunological action of nivolumab [2, 3].

Although APA development is associated with some autoimmune diseases and rheumatological disorders [4], it was also reported to be associated with certain medications, like phenytoin, hydralazine, and procainamide [5, 6]. Antiphospholipid antibodies were detected in 18/30 patients with melanoma who received interleukin-2 (IL-2) and/or alpha-interferon (*α*-IF) [7]. Development of APA with anti-PD-1 was not reported.

## 2. Case Presentation

After receiving a third dose of nivolumab, a 71-year-old Caucasian male with a known history of malignant melanoma presented to the emergency department with increasing fatigue, dizziness, exertional dyspnoea, and decreased urine output. He reported no fever, chest pain, cough, or headache. He was afebrile and hemodynamically stable. He had hepatomegaly but no splenomegaly or ascites.

He had a medical history of hypertension, depression, and hypothyroidism. He had never smoked tobacco, did not drink alcohol, and used no recreational drugs. He was not married. His father had gastric cancer, and his sister had uterine cancer. Home medications included ondansetron, enalapril, levothyroxine, and sertraline.

The patient was diagnosed with malignant melanoma of the left side of the upper back four months earlier by biopsy; he underwent wide excision and sentinel lymph node dissection. PET scan was abnormal for osseous metastasis to C7 and L1 vertebral bodies and metastatic hypermetabolic lymph nodes in the left chest wall and axilla.

Subsequently, the patient was started on nivolumab 240 mg and received a total of 3 doses; the last one was 5 days before admission. On admission, he was found to have anaemia, thrombocytopenia, and elevated liver enzymes. In addition, he was found to have prolonged prothrombin time and activated partial thromboplastin time.

## 3. Investigations

### 3.1. Haematology

Hemoglobin (Hb) 8.6 g/dL, white blood cell (WBC) count 5.4 K/uL, and platelet (Plt) count 44 K/uL.

### 3.2. Chemistry

Creatinine 1.0 mg/dL, calcium 7.2 mg/dL, alanine aminotransferase (ALT) 57 U/L, aspartate aminotransferase (AST) 110 UL, albumin 2.8 mg/dL, and total bilirubin 1.3 Mg/dL.

### 3.3. Coagulation Profile

Prothrombin time (PT) 23 seconds, INR 1.99, activated partial thromboplastin time (aPTT) > 235 seconds, aPTT 1 : 1 mix 52 seconds (prolonged), fibrinogen level 228 mg/dL (normal range 193–473), factor IX assay 190%, factor VIII assay 540% (normal range 50–150%), factor IX assay 190% (normal range 50–150%), and factor XI assay 51% (normal range 50–150%). Inhibitor screen results are suggestive of a possible inhibitor.

### 3.4. Immunology

PTT lupus anticoagulant (LA) > 180  seconds and APTT 1 : 1 mix 52 seconds. Hexagonal phospholipid neutralization test is positive.

### 3.5. Imaging

Computed tomography scan (CT) of the abdomen showed multiple hepatic and splenic metastasis in addition to three pulmonary nodules (metastasis).

## 4. Differential Diagnosis

Differential diagnosis included APA development induced by nivolumab, liver disease related coagulopathy, and melanoma-related coagulopathy (disseminated intravascular coagulopathy (DIC)).

## 5. Treatment

The patient was treated with vitamin K 5 mg orally daily for 4 days, prednisone 60 mg orally daily for 4 days and then for 7 days after discharge, and intravenous sodium chloride (IVF).

## 6. Outcome and Follow-Up

The patient's symptoms improved after receiving IVF; however, his PT and aPTT did not improve with vitamin K supplementation or prednisone. His Hb and WBCs remained low but did not require any transfusions. He did not develop deep vein thrombosis or arterial thrombosis. His liver enzymes remained elevated. The patient was discharged home after 4 days in a stable condition. He chose not to be resuscitated and expired a few days later at his house.

## 7. Discussion

Nivolumab and pembrolizumab are monoclonal antibodies against PD-1 that are approved for advanced melanoma. They work by inhibiting PD-1 and thus result in enhancing T-cell activity against the tumor [1]. PD-1 is also found on B-cell surface. Adverse events like colitis, hypothyroidism, and arthropathies have been reported and potential relation to the activated immune system has been proposed, as these adverse events were successfully treated with immunosuppressants such as steroids and infliximab [1].

We believe our patient developed the APA as a result of immune activation caused by nivolumab. This phenomenon was reported before; the association of APA and some autoimmune diseases like systemic lupus erythromatosis and rheumatoid arthritis is well reported [4]. In addition, the development of APA was observed in 18 out of 30 patients with advanced melanoma who received immunotherapy (*α*-interferon and/or IL-2) [7]. Ipilimumab, a monoclonal antibody against cytotoxic T-lymphocyte-associated antigen 4, was reported to cause acquired haemophilia A with development of antifactor VIII inhibitor [8]. The association between melanoma and APA development was suggested by a study that found one patient (out of 97 screened) with melanoma and APA (the patient did not receive immunotherapy), but it has not been observed in any other study according to our knowledge [9]. Our patient did not have the clinical manifestations associated with APA syndrome (deep vein thrombosis and/or arterial thrombosis). The development of APA can increase the risk of venous thromboembolism, especially when other risk factors are present (active malignancy or pregnancy) [10].

Melanoma and other malignant tumor can induce coagulopathy through DIC; however, our patient did not have schistocytes on blood film and his fibrinogen level was within normal range. Prolonged PT and aPTT can be seen with liver disease as well (our patient had liver metastasis) mainly through decreased production of coagulation factors. However, his other coagulation factors (fibrinogen, factor IX, and factor XI) were within normal range, and his aPTT did not correct after mixing studies, suggesting an inhibitor mechanism of coagulopathy rather than decreased production.

## 8. Learning Points/Take Home Messages

1. Nivolumab may induce the development of APA; physicians should be aware of this reported adverse event and test for it.
2. Physicians should observe for more immune-related adverse events with increasing use of nivolumab.
3. Treatment for immune-mediated adverse events of nivolumab should include an immunosuppressant.
